# Endoplasmic reticulum stress activates transglutaminase 2 leading to protein aggregation

**DOI:** 10.3892/ijmm.2014.1640

**Published:** 2014-01-30

**Authors:** JIN-HAENG LEE, JAEHO JEONG, EUI MAN JEONG, SUNG-YUP CHO, JEONG WOOK KANG, JISUN LIM, JINBEOM HEO, HYUNSOOK KANG, IN-GYU KIM, DONG-MYUNG SHIN

**Affiliations:** 1 Department of Biochemistry and Molecular Biology, Seoul National University College of Medicine, Seoul, Republic of Korea; 2 Department of Biomedical Sciences, Asan Medical Center, University of Ulsan College of Medicine, Seoul, Republic of Korea; 3 Department of Physiology, Asan Medical Center, University of Ulsan College of Medicine, Seoul, Republic of Korea

**Keywords:** unfolded protein response, transglutaminase 2, αB-crystallin, protein aggregation, HLE-B3

## Abstract

Aberrant activation of transglutaminase 2 (TGase2) contributes to a variety of protein conformational disorders such as neurodegenerative diseases and age-related cataracts. The accumulation of improperly folded proteins in the endoplasmic reticulum (ER) triggers the unfolded protein response (UPR), which promotes either repair or degradation of the damaged proteins. Inadequate UPR results in protein aggregation that may contribute to the development of age-related degenerative diseases. TGase2 is a calcium-dependent enzyme that irreversibly modifies proteins by forming cross-linked protein aggregates. Intracellular TGase2 is activated by oxidative stress which generates large quantities of unfolded proteins. However, the relationship between TGase2 activity and UPR has not yet been established. In the present study, we demonstrated that ER stress activated TGase2 in various cell types. TGase2 activation was dependent on the ER stress-induced increase in the intracellular calcium ion concentration but not on the TGase2 protein expression level. Enzyme substrate analysis revealed that TGase2-mediated protein modification promoted protein aggregation concurrently with decreasing water solubility. Moreover, treatment with KCC009, a TGase2 inhibitor, abrogated ER stress-induced TGase2 activation and subsequent protein aggregation. However, TGase2 activation had no effect on ER stress-induced cell death. These results demonstrate that the accumulation of misfolded proteins activates TGase2, which further accelerates the formation of protein aggregates. Therefore, we suggest that inhibition of TGase2 may be a novel strategy by which to prevent the protein aggregation in age-related degenerative diseases.

## Introduction

Aberrant accumulation of protein aggregates is a key characteristic of several age-related degenerative disorders (1). During life, cells are chronically exposed to oxidative stress resulting from mitochondrial inefficiency or dysfunction, which can cause a variety of oxidative modifications to proteins such as thiolation, glycation, phosphorylation and deamidation (2). The oxidatively damaged proteins are prone to form into tangled aggregates by non-enzymatic reaction unless they are degraded by the ubiquitin-proteasome system in a timely manner (3).

Transglutaminase 2 (TGase2) is a member of a family of enzymes that post-translationally modify proteins by catalyzing an acyl transfer reaction between the γ-carboxamide group of protein glutamine residues and the ɛ-amino group of lysine residues (protein cross-linking), or polyamines (protein polyamination) (4). The transamidation activity of TGase2 is calcium-dependent and produces irreversible protein polymers that are resistant to proteolytic degradation (5). Thus, TGase2 plays a crucial role in the formation of insoluble protein aggregates and has been implicated in the pathogenesis of many diseases termed ‘conformational diseases’ (4). For example, TGase2 may contribute to the formation of crystallin polymers in age-related cataracts (6), the aggregation of huntingtin protein with an expanded polyglutamine domain as occurs in Huntington’s disease (7), and the accumulation of insoluble neurofibrillary tangles and β-amyloid plaques in Alzheimer’s disease (8,9). We recently demonstrated that reactive oxygen species (ROS) activate intracellular TGase2 in various cell types (10) and that the transforming growth factor β (TGFβ) signaling pathway is involved in TGase2 activation (11). However, the role of TGase2 in cellular responses to other stressors remain to be elucidated.

The accumulation of misfolded proteins in the endoplasmic reticulum (ER) can trigger a specific stress response called the unfolded protein response (UPR) (12). Accordingly, several chemical inhibitors of protein folding procedures such as tunicamycin (TM; inhibitor of N-glycosylation), dithiothreitol (DTT) or β-mercaptoethanol (β-ME; permeable reducing agents), and thapsigargin (TG; inhibitor of the Ca^2+^ pump in ER) induce the UPR response. The UPR pathway is important for regulation of normal cellular homeostasis and may also play key roles in the pathology of conformational diseases (13). Cells can employ different UPR programs depending on the level of ER stress (14). In response to moderate stress, cells reduce the cellular burden of improperly folded proteins by attenuating *de novo* protein synthesis through phosphorylation of the protein translation initiation factor 2 (eIF2α) and by inducing the expression of chaperone proteins, including several glucose response proteins (GRPs). By contrast, sustained and unresolved ER stress may trigger programmed cell death through activation of activating transcription factor 4 (ATF4), ATF6, CCAAT/enhance-binding protein homologous protein (CHOP) and caspases (12,13,15,16). Moreover, at the cellular level, ER stress induces an increase in intracellular Ca^+^ concentration and ROS generation (14,17). Of note, these intracellular conditions are known to activate *in situ* transamidation activity of TGase2 (10,11,18,19), suggesting that the aggregate formation of misfolded proteins is accelerated by TGase2-mediated protein modifications. Thus, it is reasonable to investigate the likely relationship between protein misfolding stress and TGase2 activity. In the present study, we found that ER stress induces TGase2 activation in various cell types, including lens epithelial cells, and that the activated enzyme plays a critical role in the formation of protein aggregates.

## Materials and methods

### Cell culture

Human lens epithelial (HLE-B3), erythroleukemia (K562), cervical carcinoma (HeLa), and neuroblastoma (SH-SY5Y) cell lines were cultured as previously described (10). For UPR activators, cells were treated with culture media containing β-ME (Sigma, St. Louis, MO, USA; 7.5 mM), DTT (Sigma; 3 mM), TG (Sigma; 1 mM) or TM (Sigma; 5 μg/ml) for the indicated times and then maintained in culture until analysis. KCC009, a specific chemical inhibitor of TGase2 (20), was added at a concentration of 125 μM to inhibit transamidation activity. To differentiate SH-SY5Y cells, the cells were treated with 5 μM retinoic acid (RA) (Sigma) for 1 day before induction of ER stress.

### Measurement of intracellular calcium

Ca^2+^ levels were measured by fluorimetry using the Fluo-4AM (Molecular Probes, Carlsbad, CA, USA). Approximately 3×10^4^ cells were grown overnight in a 96-well microplate. After exposure to ER stress, the cells were incubated with 100 μl of assay buffer (Hanks’ balanced salt solution in 20 mM HEPES, pH 7.4) containing 5 μM Fluo-4AM at 37°C for 30 min and for an additional 30 min at room temperature. The cells were then washed with the assay buffer 4 times, and then the intensity of fluorescence was measured using a fluorescence microplate reader (Cary Eclipse; Varian, Palo Alto, CA, USA) with excitation set at 488 nm and emission set at 516 nm. After reading, the cells were stained with crystal violet (Sigma) to normalize the fluorescence value. Intracellular Ca^2+^ levels were expressed as the ratio of values in ER stress-exposed cells to that of untreated cells. EGTA (1.5 mM) and BAPTA-AM (20 μM, Molecular Probes) were used for calcium chelation.

### In situ transamidation assays

*In situ* TGase2 activity was measured by determining the biotinylated pentylamine (BP) incorporated into cellular proteins. Cells were incubated with 1 mM BP (Pierce, Rockford, IL, USA) for 1 h prior to harvesting, and then the cell extracts were prepared by sonication in phosphate-buffered saline (PBS) with a protease inhibitor cocktail, followed by centrifugation (14,000 × g, 10 min at 4°C). For the solid-phase microtiter plate assay, cell extracts (0.2 mg/ml, 50 μl/well) in coating buffer (50 mM Tris-Cl, pH 7.5, 150 mM NaCl, 5 mM EGTA, 5 mM EDTA) were added to each well of a 96-well microtiter plate. *In situ* TGase2 activity was evaluated by determining the incorporated BP using HRP-conjugated streptavidin (Pierce), followed by reaction with *O*-phenylenediamine dihydrochloride (Sigma). Assays were quantified by measuring the absorbance at 490 nm on a microplate spectrophotometer (Molecular Devices). *In situ* TGase2 activities were normalized by subtracting values representing endogenous biotin-conjugated proteins that were obtained without the addition of biotinylated pentylamine. *In situ* TGase2 activity was presented as folds of activation, compared to non-treated experiments. Western blot analysis was performed by subjecting the cell extracts (30 μg) to SDS-PAGE using a 12% gel, and the proteins were then transferred to nitrocellulose membranes. The proteins incorporated with BP were probed with HRP-conjugated streptavidin, followed by chemiluminescence detection (Pierce).

### Western blotting

The cell extracts (30 μg) were separated on 12% SDS-PAGE gels following preparation in RIPA lysis buffer (Santa Cruz Biotechnology, Inc., Santa Cruz, CA, USA). Protein levels were assessed by probing with monoclonal antibodies specific for TGase2 (21) and β-actin (Santa Cruz Biotechnology, Inc.). To investigate the TGase2-catalyzed crosslinking of lens proteins, whole cell extracts were prepared by sonication in homogenate buffer (50 mM Tris-Cl, pH 6.8, 6 M urea, 2% SDS, 40 mM DTT and a protease inhibitor cocktail) and further centrifuged at 12,000 × g for 10 min at 4°C. Proteins were quantified using the BCA method (Pierce), resolved on 6–15% SDS-PAGE gels, and subsequently analyzed using antibodies specific for αB-crystallin (Stressgen) and vimentin (Santa Cruz Biotechnology, Inc.). For the solubility experiments, cell extracts were separated into the water-soluble and water-insoluble fractions as previously described (11).

### Cell viability assay

Cell viability after treatment with UPR stress was determined by MTT assay (Sigma) according to the manufacturer’s protocol. The reduction of MTT reagent was quantified after 4 h by measuring the absorbance at 570 nm on a microplate spectrophotometer (Molecular Devices).

### Statistical analysis

All data on TGase2 activity and cell viability were analyzed using one-way or two-way ANOVA with Bonferroni post-tests. All analyses were performed using GraphPad Prism 5.0 statistical software (GraphPad Software, La Jolla, CA, USA). Statistical significance was defined as p<0.05 or p<0.01.

## Results

**Figure 1:**
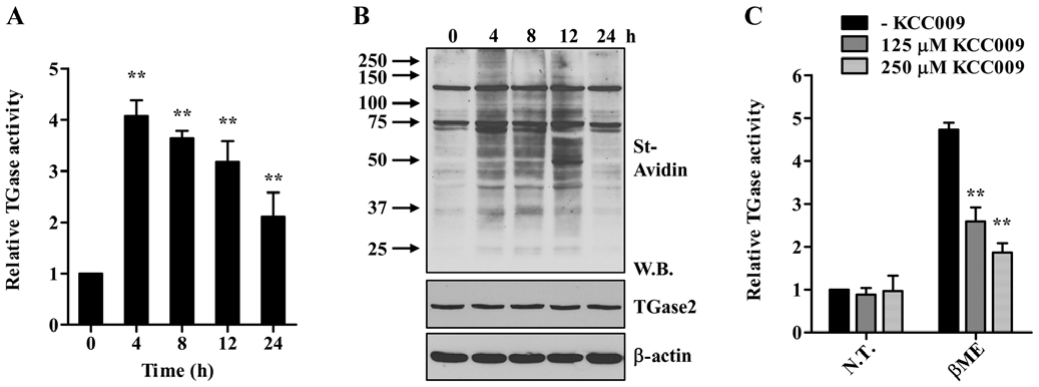
Treatment with β-ME activates TGase2 in HLE-B3 cells. (A and B) HLE-B3 cells were exposed to β-ME (7.5 mM) for the indicated times. Cells were incubated for 1 h with BP (1 mM), and intracellular TGase2 activity was determined by microtiter plate assay (A) and western blot analysis (B). Streptavidin-HRP (St-Avidin) was used to detect the BP incorporated into the proteins. ^**^p<0.01 compared to 0 h. (C) *In situ* TGase2 activity in HLE-B3 cells exposed to β-ME for 4 h in the absence or presence of the indicated concentrations of KCC009. Relative TGase2 activity is expressed as the fold-change compared with the values for non-treated cells. Results are presented as means ± SD (n=3). ^**^p<0.01 compared to cells in the absence of KCC009 (two-way ANOVA with Bonferroni post-test). β-ME, β-mercaptoethanol; BP, biotinylated pentylamine; W.B., western blot; N.T., not treated.

The aberrant activation of TGase2 accelerates the pathological misfolding/aggregation of proteins (10,11). To test whether ER stress activates TGase2, we measured the intracellular transamidation activity in the HLE-B3 cells following treatment with β-ME. *In situ* activity of TGase2 was monitored by incubating the cells with BP and by measuring the BP-incorporated proteins in the cell extracts using a well plate or SDS-PAGE assay. As shown in Fig. 1A and B, treatment with β-ME significantly increased intracellular TGase2 activity, peaking at 4 h after treatment. The level of TGase2 protein was little affected under this condition (Fig. 1B), suggesting that latent TGase2 present under normal culture conditions was activated by ER stress as previously observed in the case of the oxidative stress (10,11). In addition, treatment with KCC009, a chemical inhibitor for TGase2 abrogated the β-ME-induced increase of TGase2 activity (Fig. 1C).

**Figure 2:**
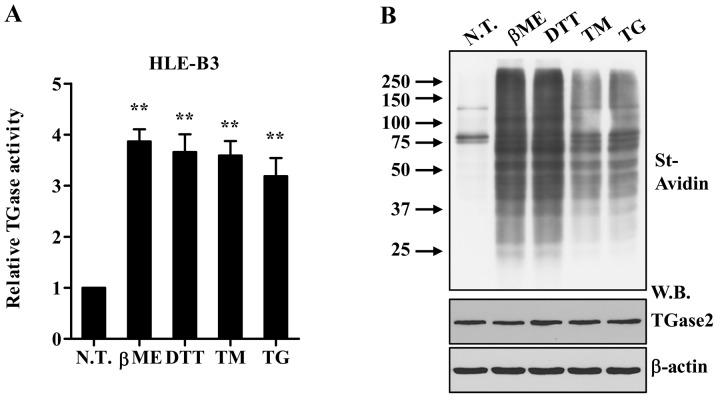
Various ER stress-causing agents activate TGase2 in HLE-B3 cells. (A and B) *In situ* TGase2 activity in HLE-B3 cells exposed to β-ME (7.5 mM) or DTT (3 mM) for 4 h and TG (1 mM) or TM (5 μg/ml) for 24 h. Enzymatic activity was determined by microtiter plate assay (A) and western blot analysis (B). ^**^p<0.01 compared to N.T. N.T., not treated; β-ME, β-mercaptoethanol; DTT, dithiothreitol; TM, tunicamycin; TG, thapsigargin; W.B. western blot.

**Figure 3:**
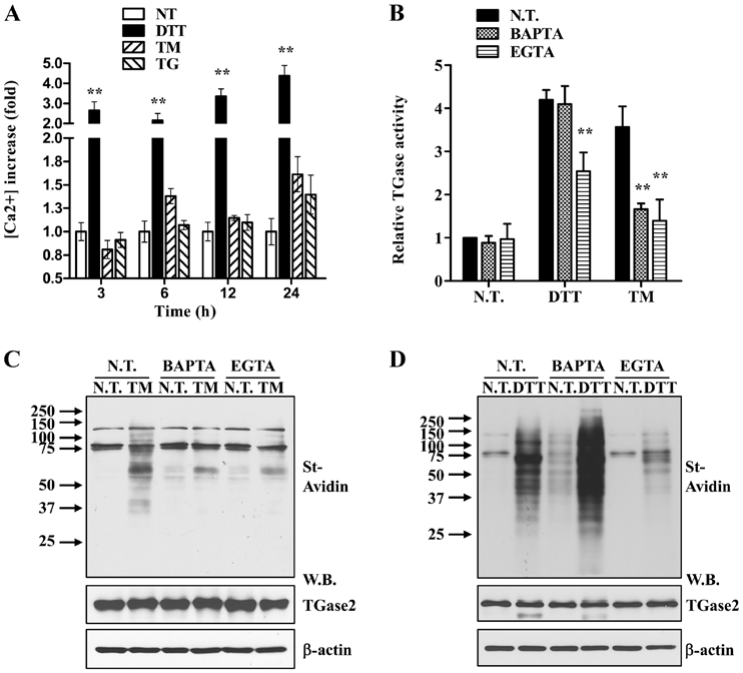
ER stress activates TGase2 through an increase in intracellular calcium. (A) Increase in [Ca^2+^]_i_ in response to ER stress. Data are expressed as the means ± SD (n=6). ^**^p<0.01 compared to N.T. (two-way ANOVA with Bonferroni post-test). (B) Intracellular TGase2 activity was measured in HLE-B3 cells following exposure to 5 μg/ml TM or DTT for 24 h in the presence or absence of either EGTA (1.5 mM) or BAPTA-AM (20 μM). (C and D) Equal amounts of whole cell extracts were immunoblotted with HRP-conjugated streptavidin (St-Avidin) (upper panel) and antibodies specific for TGase2 and β-actin, respectively (lower panel). N.T., not treated; DTT, dithiothreitol; TM, tunicamycin; TG, thapsigargin; W.B., western blot.

We next evaluated whether other ER stress-causing agents affect the TGase2 activity in HLE-B3 cells. As shown in Fig. 2, *in situ* TGase2 activity was similarly increased with little change in the protein levels following treatment with most UPR activators, including DTT, TG, and TM. Supra-physiological stressors, such as DTT and β-ME, rapidly increased the TGase2 activity at ~4 h. By contrast, treatment with TG or TM reached a peak of induced enzyme activity by 24 h, indicating that each ER stress exhibited distinct TGase2 activation kinetics. Since the transamidation activity of TGase2 is calcium-dependent (22), intracellular calcium concentrations were measured following treatment with different ER stress inducers. In correlation with the observed time-dependent rise in TGase2 activity, Ca^+^ concentrations in HLE-B3 cells rapidly increased with DTT, but increased more gradually over 24 h following TG or TM treatment (Fig. 3A). In addition, Ca^2+^ chelation by BAPTA-AM or EGTA abrogated the effect of TM on TGase2 activity (Fig. 3B and C), indicating that an elevated intracellular Ca^2+^ concentration is required for enzyme activation. The increased TGase2 activity stimulated by DTT was partially attenuated by the presence of EGTA; however it was little affected by treatment with BAPTA-AM (Fig. 3B and D). The TGase2 protein level was also similar in all of the conditions tested. Therefore, these results demonstrate that UPR increases intracellular calcium ion concentrations that activate TGase2.

**Figure 4:**
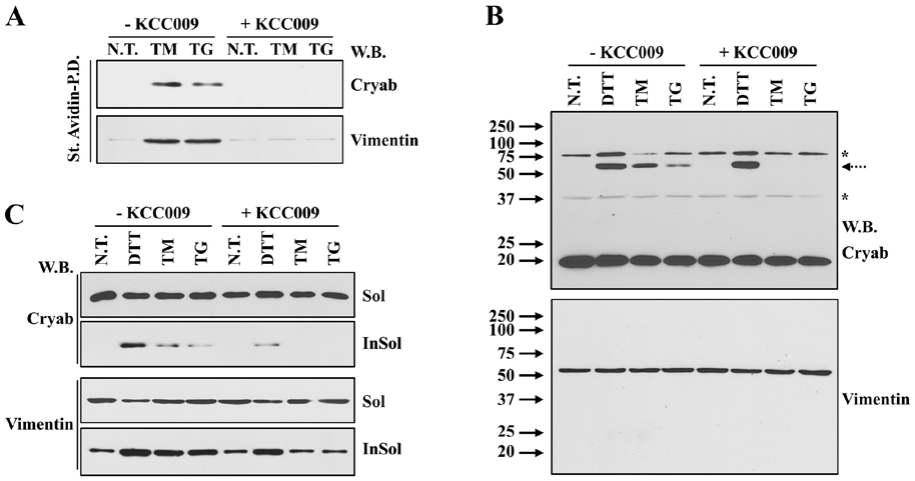
TGase2 mediates UPR-induced protein aggregation. (A) HLE-B3 cells were treated with TG (1 mM) or TM (5 μg/ml) for 24 h in the presence or absence of KCC009 (125 μM). Proteins incorporated with BP were separated using streptavidin and subjected to western blot analysis. (B) Cross-linking of αB-crystallin in whole extracts from cells exposed to UPR stress. Arrow and asterisk (*) indicate the cross-linked αB-crystallin and non-specific bands, respectively. (C) The solubility of modified αB-crystallin and vimentin was assessed by separating cell extracts into soluble (Sol) and insoluble (InSol) fractions. N.T., not treated; TM, tunicamycin; TG, thapsigargin; W.B., western blot; Cryab, αB-crystallin; DTT, dithiothreitol.

To gain insight into the potential pathophysiological roles of TGase2, we evaluated whether TGase2 modifies individual lens proteins upon ER stress. To address this issue, proteins cross-linked with BP by TGase2 were isolated using streptavidin pull-down and subjected to western blot analysis. BP modification of αB-crystallin and vimentin was observed specifically in cells treated with TM or TG, but it was inhibited by treatment with KCC009 (Fig. 4A). Previous studies report that the lens proteins modified by TGase2 in response to oxidative stress or TGFβ stimulation were dimerized and showed decreased water solubility (10,11). Similarly, treatment with each UPR activator tested led to the formation of high-molecular-weight protein of αB crystallin, but not vimentin (Fig. 4B). When the cell extracts were separated into water soluble and insoluble fractions, both αB crystallin and vimentin proteins in the water-insoluble fraction increased in the cells exposed to ER stress (Fig. 4C). Moreover, KCC009 significantly inhibited the protein cross-linking and water insolubility of lens proteins stimulated by treatment with TG and TM, but not DTT (Fig. 4B and C). These results demonstrate that the activation of TGase2 under ER stress conditions plays a pivotal role in the formation of water-insoluble aggregates of lens proteins.

**Figure 5:**
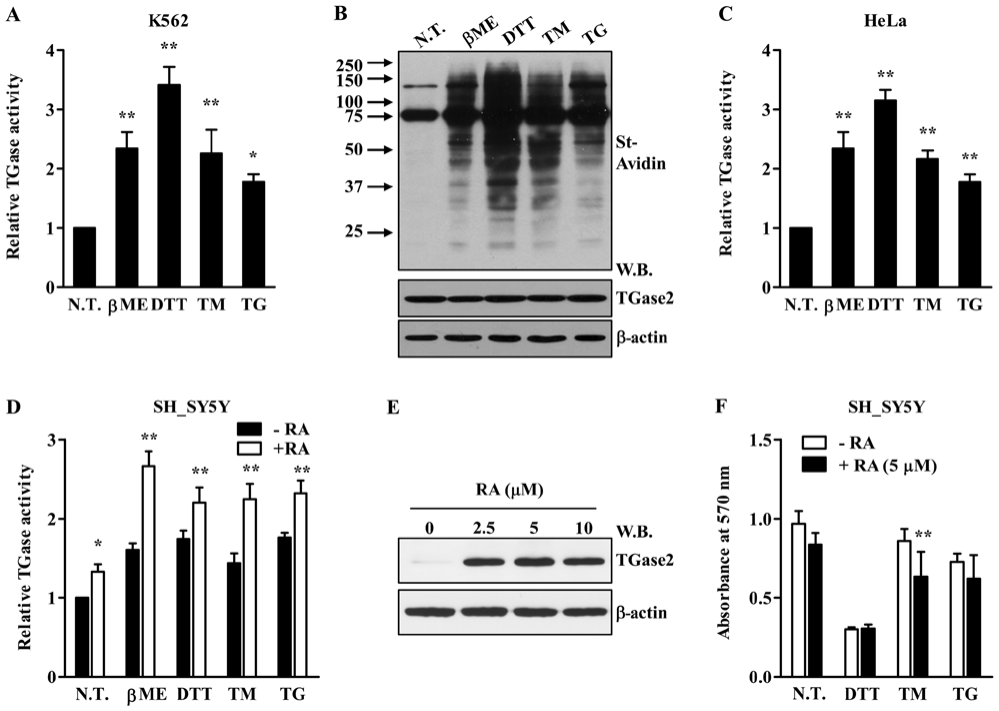
ER stress activates TGase2 in various cell types. (A–D) *In situ* TGase2 activity in the indicated cell lines exposed to β-ME (7.5 mM) or DTT (3 mM) for 4 h and TG (1 mM) or TM (5 μg/ml) for 24 h. Relative TGase2 activity is expressed as the fold-change compared to values for non-treated cells, presented as means ± SD (n=3). ^**^p<0.01 compared to N.T. (E and F) Western blot analysis of TGase2 (E) and cell viability (F) of SH-SY5Y cells following treatment with retinoic acid (RA). Reduction of the MTT reagent was quantified by measuring the absorbance at 570 nm. ^**^p<0.01 compared to cells in the absence of RA (two-way ANOVA with Bonferroni post-test). N.T., not treated; β-ME, β-mercaptoethanol; DTT, dithiothreitol; TM, tunicamycin; TG, thapsigargin; St-Avidin, HRP-conjugated streptavidin; W.B., western blot.

Next, we investigated whether ER stress activates TGase2 in other types of cells. As shown in Fig. 5, ER stress inducers increased TGase2 intracellular activity in most of the cell types evaluated, including human erythrocarcinoma (K562; Fig. 5A and B), cervical cancer (HeLa; Fig. 5C) and neuroblastoma (SH-SY5Y; Fig. 5D) cell lines. These results indicate that the activation of TGase2 is not specific to lens epithelial cells. Moreover, treatment of SH-SY5Y cells with retinoic acid (RA) induced the expression of TGase2, and it may play an important role in cell survival (23–25). When SH-SY5Y cells were pre-treated with RA, the protein level of TGase2 increased (Fig. 5E). Under this condition, ER stress further increased *in situ* TGase activity in SH-SY5Y cells (Fig. 5D). When cell viability was evaluated following exposure to ER stress, little significant difference in cell death was noted between non-treated and RA-treated SH-SY5Y cells (Fig. 5F), suggesting that UPR-induced cell death was independent of TGase2-mediated protein aggregation.

## Discussion

The present study demonstrated that ER stress activates transglutaminase 2 (TGase2) in several cell types which subsequently plays a causal role in the formation and accumulation of intracellular protein aggregates. The TGase enzyme family consists of 8 enzymes, TGase1 to TGase7, and Factor XIIIa. Each TGase isoenzyme shows a restricted pattern of tissue distribution where it plays a specific function, such as the formation of the barrier structure in skin (TGase1, TGase3 and TGase5), formation of fibrin aggregates in blood clots (coagulation Factor XIIIa), and generation of the post-coital plug of seminal fluid (TGase4) (26–28). By contrast, TGase2 is unique among the TGase family members in its ubiquitous tissue expression and widespread subcellular localization (22). In the lens epithelium, TGase2 is the major TGase isoform and was found to induce the formation of lens protein aggregates in response to UV-irradiation and oxidative stress in a lens organ culture model of cataract (11,29). In the present study, we showed that several of the ER stress-causing agents tested increased the *in situ* transamidation reaction in lens epithelial cells (Figs. 1 and 2). Moreover, ER stress-induced protein aggregation was reduced by treatment with a chemical inhibitor for TGase2 (Fig. 1C). These results demonstrate that unfolded or misfolded proteins produced by oxidative stress activate TGase2 leading in the accumulation of protein aggregates.

Previous studies have shown that intracellular TGase2 activity does not correlate with its protein expression level and that TGase2 activation is cell type-dependent (10). Under normal culture conditions, intracellular TGase2 activity was not observed in lens epithelial cells despite of the high TGase2 protein level (Figs. 1 and 2), indicating that control of this enzyme is tightly regulated in the intracellular environment. The transamidation reaction of TGase2 is dependent on the intracellular calcium level (30). UPR activators evaluated here increased the intracellular concentration of Ca^2+^, and Ca^2+^ chelation prevented TGase2 activation by UPR (Fig. 3A and B), indicating that the rise in intracellular Ca^2+^ could explain the observed increase in TGase2 activity despite of little change in its protein level. However, DTT treatment activated TGase2 even in the presence of BAPTA-AM (Fig. 3B and C), suggesting that other cellular factor(s) may be involved in the regulation of TGase2 activity in response to DTT treatment.

In the present study, we employed a lens epithelial cell line to investigate the role of TGase2 in the formation of intracellular aggregates of misfolded proteins. For this purpose, lens tissue may provide several advantages since lens epithelial cells have a much simpler protein complexity and accumulate oxidatively modified proteins without degradation (31). A single layer of lens epithelial cells differentiates into fiber cells, which make up the lamellae inside the lens without replacing the older fiber cells (31). The misfolding of lens proteins such as crystallins and vimentin is easily monitored using dimerization and water-solubility as assessed by western blotting (Fig. 4). Moreover, lens protein aggregation can be easily detected by the development of lens opacity (11). Thus, *ex vivo* lens organ culture and *in vivo* animal models may be excellent systems for the development of pharmaceuticals that inhibit the protein aggregation caused by a variety of forms of cellular stress.

Our results showed that TGase2 was also activated by ER stress in other cell types including neuroblastoma cells. Of note, it is known that TGase2 post-translationally modifies several aggregation-prone proteins such as amyloid β-peptide, tau, α-synuclein and huntingtin (7–9,32). In particular, treatment with cystamine, a TGase inhibitor (33), or ablation of TGase2 (34) delays the onset of neurological symptoms and improves the life expectancy of Huntington’s disease model mice, suggesting that aberrant activation of TGase2 may be involved in the formation of insoluble aggregates in neurons through protein modification during ageing. Several studies have indicated that the induction of TGase2 during RA-mediated differentiation plays a protective role against neuroblastoma cell death following exposure to excitotoxic or inflammatory stress (23,24). However, another study reported that TGase2 enhanced β-amyloid 1–42-induced apoptosis (25). In the present study, RA pre-treatment had little effect on the viability of SH-SY5Y cells exposed to ER stress (Fig. 5F). Thus, further study on the unknown cellular function(s) of TGase2-mediated protein aggregation under stress conditions is required to precisely understand the pathophysiological role of this enzyme.

## References

[1] Hartl FU, Bracher A, Hayer-Hartl M (2011). Molecular chaperones in protein folding and proteostasis. Nature.

[2] Lin MT, Beal MF (2006). Mitochondrial dysfunction and oxidative stress in neurodegenerative diseases. Nature.

[3] Bence NF, Sampat RM, Kopito RR (2001). Impairment of the ubiquitin-proteasome system by protein aggregation. Science.

[4] Lorand L, Graham RM (2003). Transglutaminases: crosslinking enzymes with pleiotropic functions. Nat Rev Mol Cell Biol.

[5] Klöck C, Khosla C (2012). Regulation of the activities of the mammalian transglutaminase family of enzymes. Protein Sci.

[6] Prasanna Murthy SN, Velasco PT, Lorand L (1998). Properties of purified lens transglutaminase and regulation of its transamidase/crosslinking activity by GTP. Exp Eye Res.

[7] Jeitner TM, Bogdanov MB, Matson WR (2001). Nɛ-(γ-l-Glutamyl)-l-lysine (GGEL) is increased in cerebrospinal fluid of patients with Huntington’s disease. J Neurochem.

[8] Konno T, Morii T, Hirata A, Sato S, Oiki S, Ikura K (2005). Covalent blocking of fibril formation and aggregation of intracellular amyloidgenic proteins by transglutaminase-catalyzed intramolecular cross-linking. Biochemistry.

[9] Halverson RA, Lewis J, Frausto S, Hutton M, Muma NA (2005). Tau protein is cross-linked by transglutaminase in P301L tau transgenic mice. J Neurosci.

[10] Shin DM, Jeon JH, Kim CW (2004). Cell type-specific activation of intracellular transglutaminase 2 by oxidative stress or ultraviolet irradiation: implications of transglutaminase 2 in age-related cataractogenesis. J Biol Chem.

[11] Shin D-M, Jeon J-H, Kim C-W (2008). TGFβ mediates activation of transglutaminase 2 in response to oxidative stress that leads to protein aggregation. FASEB J.

[12] Moore KA, Hollien J (2012). The unfolded protein response in secretory cell function. Annu Rev Genet.

[13] Lin JH, Walter P, Yen TS (2008). Endoplasmic reticulum stress in disease pathogenesis. Annu Rev Pathol.

[14] Schröder M, Kaufman RJ (2005). The mammalian unfolded protein response. Annu Rev Biochem.

[15] Xin W, Li X, Lu X, Niu K, Cai J (2011). Involvement of endoplasmic reticulum stress-associated apoptosis in a heart failure model induced by chronic myocardial ischemia. Int J Mol Med.

[16] Wu LF, Wei BL, Guo YT (2012). Apoptosis induced by adenosine involves endoplasmic reticulum stress in EC109 cells. Int J Mol Med.

[17] Harding HP, Zhang Y, Zeng H (2003). An integrated stress response regulates amino acid metabolism and resistance to oxidative stress. Mol Cell.

[18] Cho S-Y, Lee J-H, Bae H-D (2010). Transglutaminase 2 inhibits apoptosis induced by calcium overload through down-regulation of Bax. Exp Mol Med.

[19] Jang GY, Jeon JH, Cho SY (2009). Transglutaminase 2 suppresses apoptosis by modulating caspase 3 and NF-kappaB activity in hypoxic tumor cells. Oncogene.

[20] Choi K, Siegel M, Piper JL (2005). Chemistry and biology of dihydroisoxazole derivatives: selective inhibitors of human transglutaminase 2. Chem Biol.

[21] Jeon JH, Choi KH, Cho SY (2003). Transglutaminase 2 inhibits Rb binding of human papillomavirus E7 by incorporating polyamine. EMBO J.

[22] Fesus L, Piacentini M (2002). Transglutaminase 2: an enigmatic enzyme with diverse functions. Trends Biochem Sci.

[23] Caccamo D, Condello S, Ferlazzo N, Currò M, Griffin M, Ientile R (2013). Transglutaminase 2 interaction with small heat shock proteins mediate cell survival upon excitotoxic stress. Amino Acids.

[24] Kweon SM, Lee ZW, Yi SJ (2004). Protective role of tissue transglutaminase in the cell death induced by TNF-alpha in SH-SY5Y neuroblastoma cells. J Biochem Mol Biol.

[25] Wakshlag JJ, Antonyak MA, Boehm JE, Boehm K, Cerione RA (2006). Effects of tissue transglutaminase on beta-amyloid1–42-induced apoptosis. Protein J.

[26] Candi E, Schmidt R, Melino G (2005). The cornified envelope: a model of cell death in the skin. Nat Rev Mol Cell Biol.

[27] Muszbek L, Bereczky Z, Bagoly Z, Komáromi I, Katona É (2011). Factor XIII: a coagulation factor with multiple plasmatic and cellular functions. Physiol Rev.

[28] Mukherjee DC, Agrawal AK, Manjunath R, Mukherjee AB (1983). Suppression of epididymal sperm antigenicity in the rabbit by uteroglobin and transglutaminase in vitro. Science.

[29] Lee S-M, Jeong EM, Jeong J (2012). Cysteamine prevents the development of lens opacity in a rat model of selenite-induced cataract. Invest Ophthalmol Vis Sci.

[30] Király R, Demény M, Fésüs L (2011). Protein transamidation by transglutaminase 2 in cells: a disputed Ca^2+^-dependent action of a multifunctional protein. FEBS J.

[31] Sue Menko A (2002). Lens epithelial cell differentiation. Exp Eye Res.

[32] Junn E, Ronchetti RD, Quezado MM, Kim SY, Mouradian MM (2003). Tissue transglutaminase-induced aggregation of alpha-synuclein: implications for Lewy body formation in Parkinson’s disease and dementia with Lewy bodies. Proc Natl Acad Sci USA.

[33] Karpuj MV, Becher MW, Springer JE (2002). Prolonged survival and decreased abnormal movements in transgenic model of Huntington disease, with administration of the transglutaminase inhibitor cystamine. Nat Med.

[34] Mastroberardino PG, Iannicola C, Nardacci R (2002). ‘Tissue’ transglutaminase ablation reduces neuronal death and prolongs survival in a mouse model of Huntington’s disease. Cell Death Differ.

